# Outcome of L-DEP regimen for treatment of pediatric chronic active Epstein–Barr virus infection

**DOI:** 10.1186/s13023-021-01909-y

**Published:** 2021-06-10

**Authors:** Honghao Ma, Liping Zhang, Ang Wei, Jun Yang, Dong Wang, Qing Zhang, Yunze Zhao, Sitong Chen, Hongyun Lian, Li Zhang, Chunju Zhou, Maoquan Qin, Zhigang Li, Tianyou Wang, Rui Zhang

**Affiliations:** 1 Hematology Center, Beijing Key Laboratory of Pediatric Hematology Oncology; National Key Discipline of Pediatrics (Capital Medical University); Key Laboratory of Major Diseases in Children, Ministry of Education; Beijing Children’s Hospital, Capital Medical University, National Center for Children’s Health, Beijing, 100045 China; 2 Hematologic Disease Laboratory, Hematology Center, Beijing Key Laboratory of Pediatric Hematology Oncology; National Key Discipline of Pediatrics (Capital Medical University); Key Laboratory of Major Diseases in Children, Ministry of Education; Beijing Children’s Hospital, Capital Medical University, National Center for Children’s Health, Beijing, 100045 China; 3 Department of Pathology, Beijing Children’s Hospital, Capital Medical University, National Center for Children’s Health, Beijing, 100045 China

**Keywords:** Chronic active Epstein–Barr virus infection, L-DEP, Response, Paediatric

## Abstract

**Purpose:**

We intended to investigate the clinical features of paediatric patients with chronic active Epstein–Barr virus infection (CAEBV) and to examine the effectiveness of the L-DEP regimen before haematopoietic stem cell transplantation (HSCT).

**Methods:**

A retrospective analysis was performed on 35 patients with CAEBV at Beijing Children’s Hospital from January 2016 to January 2020. The efficacy and adverse events of the L-DEP regimen were evaluated.

**Results:**

The median age of the 35 patients was 7.0 years old (range 2.5–17.5 years). Twenty-eight patients achieved a clinical response (80.0%, 22 in clinical CR, 6 in clinical PR) after L-DEP. In terms of virological response, 7 patients (20%) were assessed as having virological CR, and 23 patients (65.7%) had virological PR. Finally, 29 patients underwent allo-HSCT. The median survival time was 18 months (2–50 months). The 3-year overall survival rates in patients treated with chemotherapy only (n = 6) and chemotherapy followed by HSCT (n = 25) were 33.3% and 75.4%, respectively. After L-DEP 1st treatment and L-DEP 2nd treatment, the EBV-DNA loads in blood and plasma were significantly reduced compared with those before chemotherapy (median: 4.29 × 10^5^ copies/ml vs. 1.84 × 10^6^ copies/ml, Mann–Whitney U: *P* = 0.0004; 5.00 × 10^2^ copies/ml vs. 3.17 × 10^3^ copies/ml, Mann–Whitney U; *P* = 0.003; 2.27 × 10^5^ copies/ml vs. 1.84 × 10^6^ copies/ml, *P* = 0.0001; 5.00 × 10^2^ copies/ml vs. 3.17 × 10^3^ copies/ml, *P* = 0.003). Compared with the liver and spleen size before chemotherapy, the size of the liver and spleen shrank significantly after L-DEP 2nd (median 3.8 cm vs. 1.9 cm, *P* = 0.003; 3.8 cm vs. 0 cm, *P* < 0.008). In addition, after L-DEP treatment, there was no difference in the clinical or virological response rate regardless of HLH status (clinical response: 77.3% vs. 84.6%, *P* = 0.689; virological response: 90.9% vs. 76.9%, *P* = 0.337).

**Conclusion:**

The L-DEP regimen is an effective therapy in CAEBV for bridging to allo-HSCT**.**

## Introduction

Chronic active Epstein–Barr virus infection (CAEBV) is a rare lymphoproliferative disorder (LPD) that typically presents as persistent infectious mononucleosis-like disease and/or haemophagocytic lymphohistiocytosis (HLH) [1–3]. This disease occurs mainly due to inflammation accompanied by EBV infection of T or NK cells. EBV-infected T or NK cells can proliferate and infiltrate clonally into multiple organs, leading to different clinical behaviours from indolent disease to rapidly life-threatening disease. The clinical manifestations include fever, skin rashes, hepatomegaly, splenomegaly, lymphadenopathy, liver dysfunction, and higher EBV-DNA load in blood or plasma [3]. In terms of treatment, allogeneic HSCT (allo-HSCT) is the only curative treatment for eliminating EBV-infected T or NK cells [4]. Unfortunately, active disease conditions, which occur at the beginning of the conditioning treatment of allo-HSCT, are significantly associated with poor allo-HSCT outcomes [1, 5]. Therefore, to improve the prognosis, patients should receive chemotherapy before allo-HSCT to resolve disease activity [1].

However, the response rate of different chemotherapy regimens to CAEBV differs greatly. Many studies have proposed different chemotherapy regimens, including cooling therapy (combination of cyclosporine A, steroids and etoposide), CHOP (cyclophosphamide, doxorubicin, vincristine and prednisolone), and the combination of the two (cooling therapy, CHOP, Capizzi and ESCAP), but the total response rate of patients is less than 40% and even as low as 10% [1, 6, 7]. To improve the outcomes of CAEBV, it is indispensable to establish more effective chemotherapy.

In 2016, Wang et al. used PEG-asparaginase, liposomal doxorubicin, etoposide, and high-dose methylprednisolone (L-DEP regimen) as salvage therapy for controlling refractory EBV-related haemophagocytic lymphohistiocytosis (EBV-HLH) and achieved a significant EBV-DNA decrease and good outcome (OR: 85.7%) [8]. In 2020, Zhao et al. showed that the L-DEP regimen could also benefit paediatric patients with refractory EBV-HLH, with an OR rate of 61.5% [9]. Since both EBV-HLH and CAEBV are EBV-related LPDs, and CAEBV is often associated with HLH, we speculate that the L-DEP regimen could be beneficial to children with CAEBV.

In this study, we treated children with CAEBV with L-DEP regimens and analysed the clinical characteristics, prognostic factors, and effectiveness of the regimens. The results showed that the L-DEP regimen is an effective therapy in CAEBV for bridging to allo-HSCT.

## Patients and methods

### Patients

Retrospectively, single-centre data were collected from 35 patients with CAEBV treated at the haematology oncology centre of Beijing Children’s Hospital between January 2016 and January 2020. According to the World Health Organization (WHO) classification of 2016, children who fulfilled all of the following criteria were diagnosed with CAEBV: (1) sustained or recurrent IM-like symptoms persisting for more than 3 months; (2) elevated EBV genome load in the peripheral blood (PB) or the tissue lesion; (3) EBV infection of T or NK cells in the affected tissues or the PB; and (4) exclusion of other possible diagnoses: primary infection of EBV (infectious mononucleosis), autoimmune diseases, congenital immunodeficiencies, HIV, and other immunodeficiencies requiring immunosuppressive therapies or underlying diseases with potential immunosuppression [7]. The clinical diagnosis of HLH was based on the HLH-2004 diagnostic criteria [10].

Clinical data, including demographic characteristics, laboratory findings, treatment outcomes and mortality, were collected. Written informed consent was obtained from the parents, and the study was approved by the Institutional Review Board of Beijing Children’s Hospital, Capital Medical University. This clinical trial assessing the L-DEP regimen for CAEBV patients was retrospectively registered (Identifier: ChiCTR1900020574).

### Treatments

All of the patients were treated with L-DEP regimens after diagnosis. The L-DEP regimen included PEG-asparaginase (2000 U/m^2^, day 5), liposomal doxorubicin (25 mg/m^2^, day 1), etoposide (100 mg/m^2^ once a week, days 1, 8 and 15), and methylprednisolone (2 mg/kg, days 1–7; 1 mg/kg, days 8–14; 1 mg/kg, and tapering, days 15–21). The L-DEP regimen was used for at least 2 courses and up to 3 courses. The treatment response was evaluated after 3 weeks (L-DEP 1st) and/or 6 weeks (L-DEP 2nd) [8]. Once a patient achieved remission, allo-HSCT was recommended.

After chemotherapy, a total of 29 patients underwent allo-HSCT, and the other 6 patients did not undergo HSCT because of financial problems or disease deterioration.

### Evaluation of response to L-DEP regimen

The outcomes of the L-DEP regimen were evaluated and classified as follows. Clinical complete resolution (CR) was defined as no symptoms of inflammation, including fever, liver dysfunction, progressive skin lesions, or vasculitis, accompanied by a significant decrease in EBV-DNA. Resolution of some of the above symptoms of disease was defined as clinical partial resolution (PR) [1]. Progressive disease (PD) was defined as exacerbation of active disease or development of new findings of disease activity, and stable disease (SD) represented nonimprovement of disease activity or no findings of novel active disease [1]. In our article, we defined virological CR as a significant decrease in EBV-DNA load in both blood and plasma (< 10^2.5^ copies/ml DNA). A 50% drop in EBV-DNA load in either blood or plasma could be defined as virological PR.

### Monitoring the size of liver and spleen

We used Doppler ultrasound to monitor and evaluate the size of the liver and spleen. The value was marked according to the measured size under the costal margin. We conducted multiple evaluations, including at diagnosis, after L-DEP 1st and L-DEP 2nd, and before allo-HSCT.

### Evaluation of cytokines

Common cytokines, including IFN-γ (< 2.1 pg/ml), TNF-α (1.30–8.5 pg/ml), IL-10 (1.2–4.55 pg/ml), and IL-6 (< 2.05 pg/ml), were determined by ProcartaPlex bead-linked immunoassay. sCD25 (< 6400 pg/ml), which is often used as a signal of the inflammatory response, was determined by ELISA.

### Survival and follow-up

Overall survival (OS) was estimated from the date of diagnosis until the date of death due to any reason or last contact with the patients. The last follow-up date was February 2021.

### Statistics

The SPSS software package (IBM, Armonk, NY, USA), version 20.0, was used for all of the statistical analyses. The normality of numerical variables was evaluated using the Shapiro–Wilk test. The t-test or the Mann–Whitney test was used to determine differences between numerical variables with a normal or a skewed distribution between two groups, respectively. The chi-square test was used to determine whether there were differences in qualitative variables between groups. Survival analyses were carried out by Kaplan–Meier analysis, and differences in OS were compared with the two-tailed log-rank test. A *P* value < 0.05 was considered statistically significant. GraphPad Prism software, version 6.0 (Inc., San Diego, CA, USA), was used to draw graphs.

## Results

### Clinical characteristics of the CAEBV patients

Thirty-five patients with CAEBV were enrolled in this study. Of these patients, there were 20 girls and 15 boys, with a female-male ratio of 1.33:1. The median age was 7.0 years old (range 2.5–17.5 years). All of the patients had lymphadenopathy. Fever, splenomegaly, and hepatomegaly were also common in patients with CAEBV. We also found elevated levels of EBV-DNA copies/ml in blood and positive EBV-encoded RNA (EBER) in situ hybridization in bone marrow in all patients. In our study, 21 patients were tested for EBV-infected lymphocyte subtypes in peripheral blood. Eighteen of them had higher loads in all subtypes of lymphocytes, including 10 patients mainly infected with NK cells and 8 patients mainly infected with T cells. Two patients had higher loads only in NK cells and 1 patients had higher loads only in T cells. In addition, all of the patients also underwent histopathological biopsy and immunohistochemistry. After EBV infection, proliferating NK cells were mainly positive in 4 patients, T cells in 23 patients, and T-NK cells in 8 patients. The patients’ clinical characteristics at diagnosis are shown in Table 1.

**Table 1:** Clinical characteristics of 35 patients with CAEBV

	No. of patients (%) or median of clinical features (range)
Gender	
Male	15 (42.8%)
Female	20 (57.2%)
Median age at diagnosis (year)	7.0 (2.5–17.5)
EBV infection time before diagnosis (month)	3 (1–60)
Fever	31 (88.6%)
Splenomegaly	31 (88.6%)
Hepatomegaly	29 (82.9%)
Lymphadenopathy	35 (100%)
Skin lesions	9 (25.7%)
Coronary artery dilatation	4 (11.4%)
With HLH	22 (62.9%)
WBC (× 10^9^/l)	4.67 (0.95–12.2)
Neutrophil (× 10^9^/l)	1.89 (0.27–6.72)
Hb (g/l)	104 (83–133)
PLT (× 10^9^/l)	189 (39–515)
Albumin (g/l)	36.7 (21.6–44.8)
AST (U/l)	80.4 (13.8–1314.0)
ALT (U/l)	60.3 (9.8–1275.3)
Bilirubin (μmol/l)	10.43 (3.81–99.83)
Triglyceride (mmol/l)	1.90(0.58–4.09)
Fibrinogen (g/l)	2.12 (0.84–34.1)
Ferritin(ng/ml)	146.9 (24.7–4353.00)
NK activity (%)	15.43 (7.80–17.48)
Level of cytokines	
IFN-γ (pg/ml)	12.72 (1.62–769.91)
TNF-α (pg/ml)	2.82 (0.00–244.33)
IL-10 (pg/ml)	5.31 (1.35–103.42)
IL-6 (pg/ml)	32.9 (1.75–2500.00)
SCD25	10,831 (437.0–44,000.0)
EBV-DNA (copies/ml)	
In blood	1.84 × 10^6^ (5.00 × 10^3^–3.96 × 10^7^)
In plasma	3.17 × 10^3^ (5.00 × 10^2^–5.30 × 10^6^)

*No.* number, *EBV* Epstein–Barr virus, *WBC* white blood cell, *Hb* hemoglobin, *PLT* platelets, *AST* aspartate aminotransferase, *ALT* alanine aminotransferase, *IL* interleukin, *NK* natural killer

### Genetic characteristics

There were no patients with an affected sibling (family history). Whole exome sequencing of the mononucleated cells in peripheral blood showed that only two patients carried mutations possibly related to CAEBV: a spontaneous heterozygous mutation in PIK3CD [11] (c.3061G > A in exon 24) that was not detected in the patient’s parents; and compound heterozygous mutations in IFNGR1 [12, 13] (c.961G > A, c.85 + 5C > T).

In this study, the patient with the PIK3CD c.3061G > A mutation was an 8-year-old boy who had a history of multiple respiratory infections after birth. Clinical tests showed cellular immune deficiency. All subtypes of lymphocytes were lower than normal, including total T cells (50.7%), CD4/CD8 (0.49), total B cells (8.5%), T helper cells (14.8%), and T regulatory cells (30.4%). The number of EBV-infected T cells was increased (5.41 × 10^5^ per 10^6^ cells). He was treated with allo-HSCT with a survival period of 37 months and eventually died of severe respiratory infection after transplantation.

The patient with the IFNGR1 c.3061G > A mutation was a 4-year-old girl who had abnormal humoural immunity and cellular immunity and decreased NK activity. Thus, this mutation was possibly related to the pathogenesis of CAEBV. She was treated with allo-HSCT, with a survival period of 14 months thus far.

### Response to L-DEP regimen

We evaluated the response rate of chemotherapy to L-DEP treatment. The results showed that 28 patients achieved a clinical response (80.0%, 22 in clinical CR, 6 in clinical PR) after chemotherapy. One patient (2.9%) was found that have PD, and 6 patients (17.1%) had SD. In terms of virological response, 7 patients (20%) were assessed as having virological CR, and 23 patients (65.7%) achieved virological PR. Thus, the rates of clinical remission and virological remission were up to 80% and 85.7% in our patients with CAEBV, respectively.

**Fig. 1:**
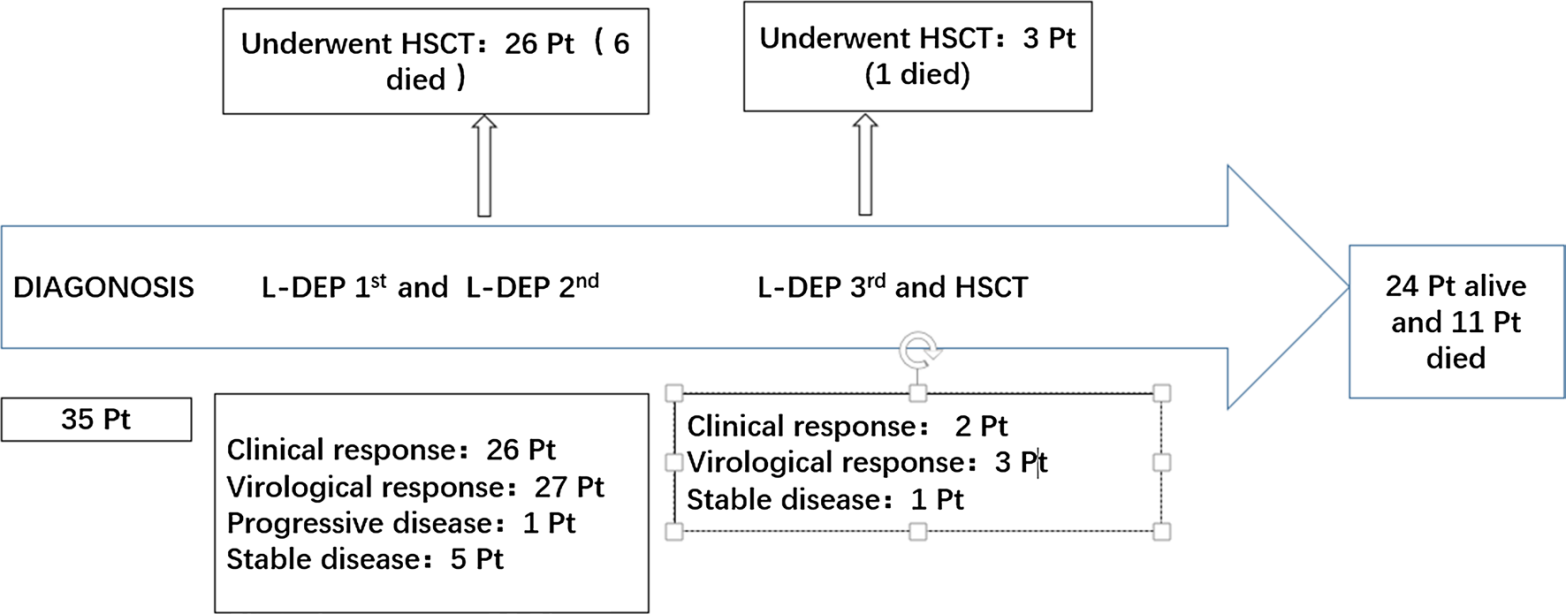
Patients’ treatment and follow-up. *Pt* patients, *L-DEP 1st* the L-DEP first course, *L-DEP 2nd* the L-DEP second course, *L-DEP 3rd* the L-DEP third course, *CR* complete response, *PR* partial response, *HSCT* hematopoietic stem cell transplantation

Ultimately, a total of 29 patients underwent allo-HSCT, and 6 patients were not treated with HSCT because of financial problems or disease deterioration. Among these patients, 11 eventually died. They died of progressive deterioration of disease before allo-HSCT (n = 4) and complications of death after transplantation (severe infection (n = 2), multiple organ failure (n = 2) and graft versus host disease (n = 3)). The median survival time was 24 months (4–57 months). Among all of the transplanted patients, 19 patients were transplanted in our transplant centre, with all of them receiving a reduced-intensity conditioning regimen. Ten patients with CAEBV chose the outside transplant centre. The conditioning regimens were combined with either myeloablative conditioning or reduced-intensity conditioning. Follow-up of the patients with CAEBV is shown in the flowchart in Fig. 1.

**Fig. 2:**
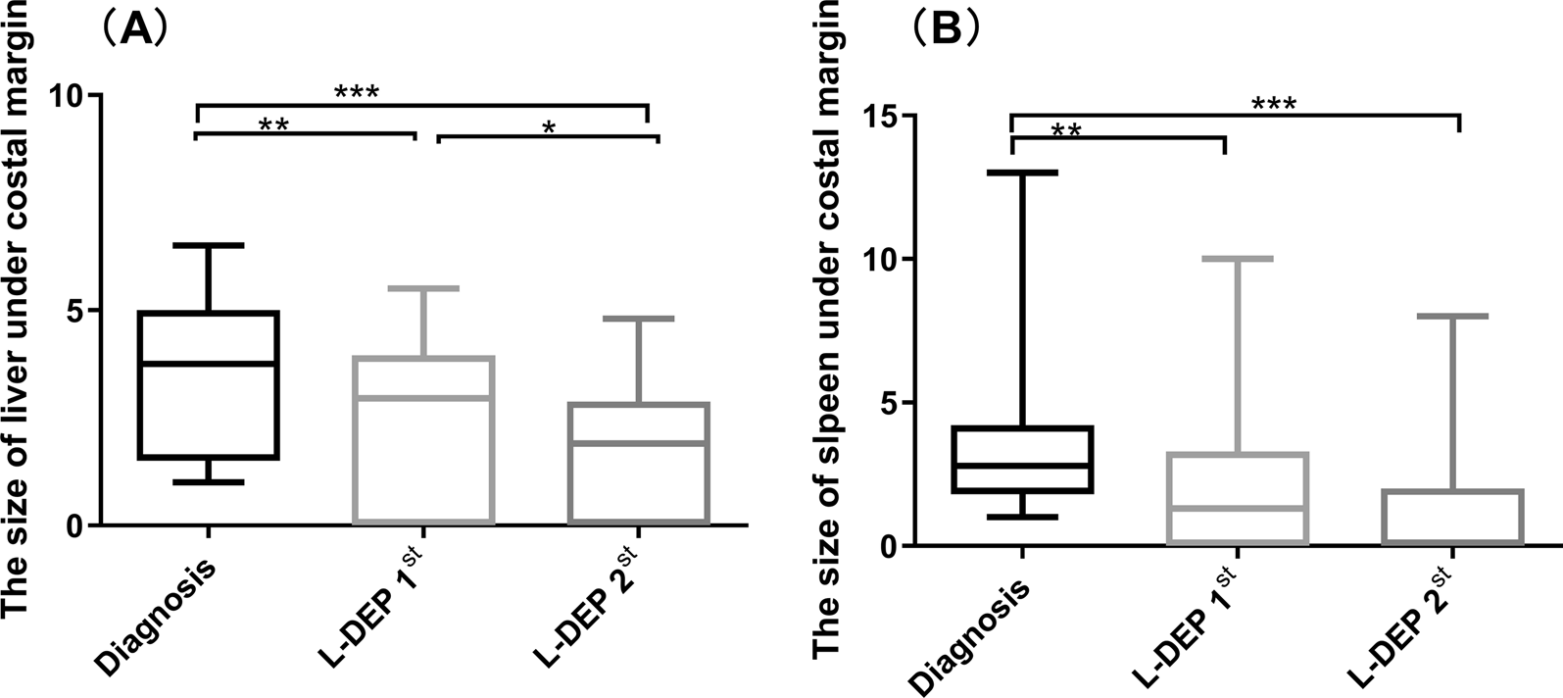
L-DEP therapy reduced the enlargements of liver (**A**) and spleen (**B**)

In addition, L-DEP 1st and L-DEP 2nd therapy significantly reduced hepatosplenomegaly: the sizes of the liver and spleen shrank from 3.8 cm (1–6.5 cm) and 2.8 cm (1.0–13.0 cm) under the costal margin at diagnosis to 2.9 cm (0–5.5 cm) and 1.3 cm (0–10.0 cm) and 1.9 cm (0–4.8 cm) and 0 cm (0–8.0 cm) after L-DEP 1st and L-DEP 2nd respectively (*P* = 0.003 and 0.007; both *P* < 0.0001, Fig. 2).

It was noteworthy that L-DEP treatment had no effect on the response rate between the groups of patients with (n = 22) or without HLH (n = 13): clinical response: 77.3% versus 84.6%, *P* = 0.689; virological response, 90.9% versus 76.9%, *P* = 0.337.

### Impact of the L-DEP regimen on EBV-DNA load in blood and plasma

All of the patients had elevated levels of EBV-DNA copies/ml in blood. After the 1st L-DEP course, the EBV-DNA loads in blood and plasma were significantly reduced compared with those before chemotherapy (blood: 4.29 × 10^5^ copies/ml vs. 1.84 × 10^6^ copies/ml, Mann–Whitney U: *P* = 0.0004; plasma: 5.00 × 10^2^ copies/ml vs. 3.17 × 10^3^ copies/ml, Mann–Whitney U: *P* = 0.003). After the 2nd L-DEP course, the load of EBV-DNA was also lower than that before chemotherapy (blood: 2.27 × 10^5^ copies/ml vs. 1.84 × 10^6^ copies/ml, Mann–Whitney U: *P* = 0.0001; plasma: 5.00 × 10^2^ copies/ml vs. 3.17 × 10^3^ copies/ml, Mann–Whitney U: *P* = 0.003), although it was similar to that after 1st L-DEP course. In addition, the EBV-DNA load in plasma became negative in 74.2% and 91.4% of patients after L-DEP 1st and 2nd respectively. Therefore, L-DEP therapy resulted in decreases in the EBV-DNA load in blood or plasma.

### Impact of L-DEP regimen on cytokine levels

**Fig. 3:**
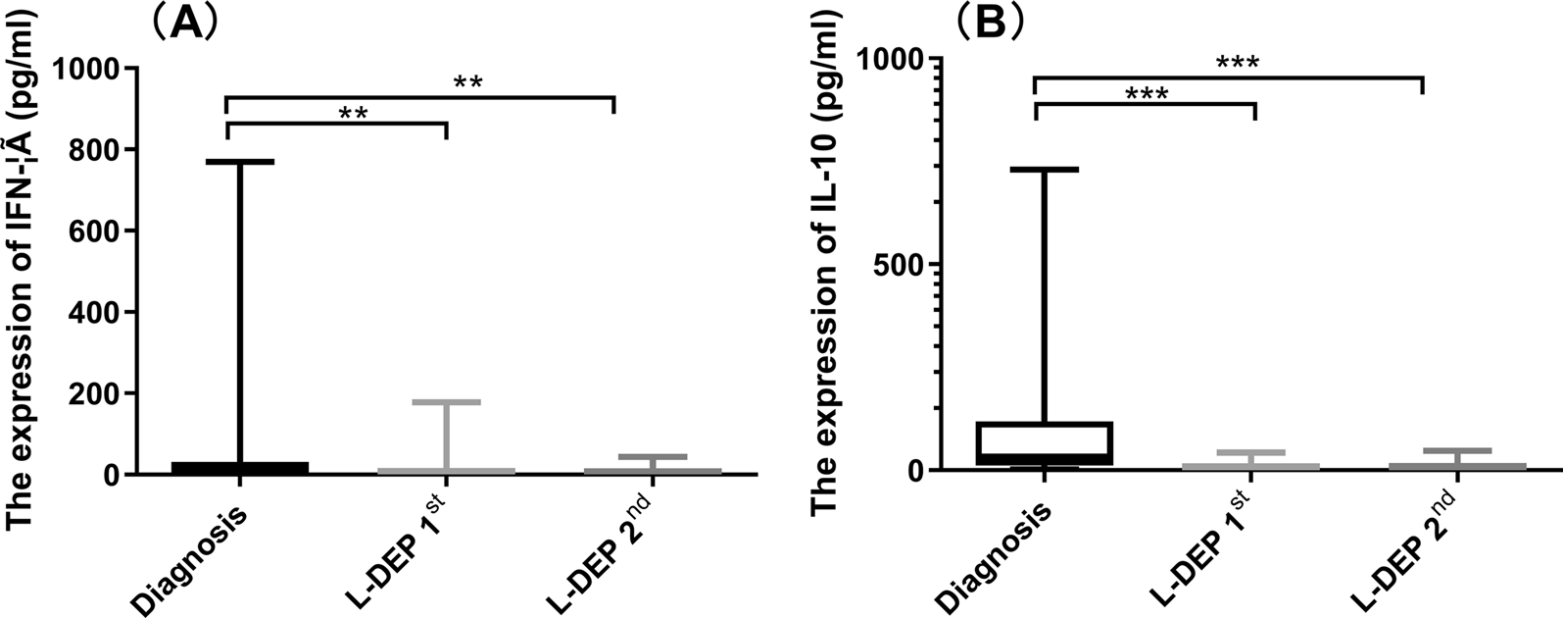
The levels of IFN-γ and IL-10 decreased obviously after L-DEP therapy. (**A**) IFN-γ, (**B**) IL-10

CAEBV is often complicated by abnormal elevation of inflammatory cytokines. As shown, L-DEP significantly decreased the levels of IFN-γ, from 61.04 pg/ml at diagnosis to 15.19 pg/ml and 7.76 pg/ml after L-DEP 1st and L-DEP 2nd, respectively (*P* = 0.015 and 0.006, respectively, Fig. 3A). The same effect was also observed in the levels of IL-10, which decreased from 116.63 pg/ml at diagnosis to 6.7 pg/ml and 7.7 pg/ml after L-DEP 1st and L-DEP 2nd, respectively (both *P* < 0.0001, Fig. 3B). However, there was no effect of L-DEP therapy on the levels of TNF-α (at diagnosis, L-DEP 1st and L-DEP 2nd: 26.74 pg/ml, 6.08 pg/ml and 4.00 pg/ml; *P* = 0.388) and IL-6 (15.43 pg/ml, 20.74 pg/ml and 16.35 pg/ml; *P* = 0.352).

We divided these patients into two groups (EBV-HLH and CAEBV group and CAEBV without HLH group) and analysed the data. We found that the IFN-γ, TNF-α, IL-10 and IL-6 levels in these two groups were not significantly different (*P* = 0.905, 0.945, 0.331, 0.759). However, TNF-α and IL-10 levels in the EBV-HLH and CAEBV groups were significantly reduced after treatment, and in the late group, IFN-γ and IL-10 were significantly reduced after treatment.

### Adverse effects of L-DEP therapy

In our patients, diarrhoea was the most common adverse effect, observed in 22 patients (62.8%). Other common adverse effects included abnormal coagulation in 17 patients (48.5%), myelosuppression in 15 patients (42.8%), high liver enzymes in 15 patients (42.8%), pancreatic injury in 14 patients (40.0%), infection in 6 patients (28.5%), and myocardial damage in 8 patients (21.0%). Acute pancreatitis and gastrointestinal bleeding were also observed in 4 patients (11.4%) and 3 patients (8.5%) respectively.

We treated these patients with symptomatic treatment, including plasma transfusion, antibiotics, glutathione, octreotide, and so on. Most of the adverse events could be alleviated and disappeared. However, 3 patients had serious treatment-related complications and died after treatment failure. Two patients with elevated liver enzymes and abnormal coagulation received plasma transfusion and glutathione therapy, but neither recovered. Both patients died of gastrointestinal bleeding and liver failure. The last patient with myelosuppression achieved virological PR but nevertheless died of uncontrolled HLH.

### Treatment outcomes

**Fig. 4:**
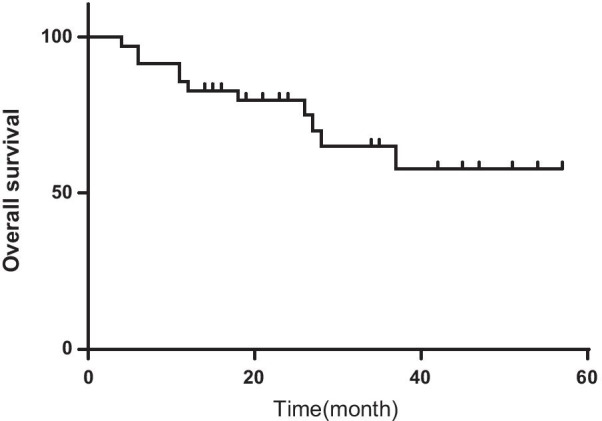
Overall survival of 35 patients with CAEBV. The probability rate of overall survival at 1-year, 3-year, and 5-year after CAEBV diagnosis were 82.9%, 65.0%, 57.8%

By the end of February 2021, 24 patients were alive, and 11 patients died. The short-term cause of death was progressive deterioration of disease before allo-HSCT (n = 4, 36.4%). The long-term causes of death (more than 2 years) were mostly due to complications posttransplantation, including severe infection (n = 2, 18.2%), multiple organ failure (n = 2, 18.2%) and graft versus host disease (n = 3, 27.3%). The median survival time was 24 months (4–57 months). The probability rates of OS at 1 year, 3 years, and 5 years were 82.9%, 79.7%, and 57.8%, respectively (Fig. 4). In addition, we found a trend toward an increase in 3-year OS in patients treated with chemotherapy only (n = 6) or chemotherapy followed by transplantation (n = 29; 33.3% vs. 75.4%, *P* = 0.098).

### Prognostic factors associated with the effectiveness of the L-DEP regimen

To analyse the prognostic factors in CAEBV, we divided the patients into two groups according to each of the common clinical and laboratory features. We used the 75th percentile or reference value of each of the numerical features as the cut-off value for grouping. However, a correlation of treatment outcome was not observed with age (≥ 4.5 years old), time from EBV infection to diagnosis (≥ 12 months), size of liver and spleen, EBV-DNA load in blood and plasma, chemotherapy (≥ 3 course), clinical CR/PR or virological CR/PR complicated by HLH, or cytokine level (see Table 2).

**Table 2:** Analysis of putative prognostic factors in CAEBV patients

Variables	Univariate analysis	
	Hazard ratio (95% CI)	*P* value
Age (≥ 4.5 years)	4.72 (0.82–10.68)	0.097
Time of EBV infection to diagnosis (≥ 12 months)	1.56 (0.39–7.80)	0.485
Liver (≥ 3.9 cm)	0.76 (0.18–3.19)	0.726
Spleen (≥ 4.1 cm)	1.38 (0.38–5.19)	0.602
AST (≥ 150 U/l)	0.64 (0.17–2.59)	0.570
ALT (≥ 100 U/l)	0.64 (0.17–2.60)	0.567
Fib (< 1.5 g/l)	0.51 (0.11–2.88)	0.511
Complicated with HLH	1.10 (0.32–3.70)	0.878
EBV-DNA in blood (≥ 5.0 × 10^6^ copies/ml)	0.93 (0.25–3.44)	0.918
EBV-DNA in plasma (≥ 1.0 × 10^5^ copies/ml)	1.15 (0.23–5.83)	0.850
Chemotherapy (≥ 3 course)	0.31 (0.04–2.07)	0.235
Clinical CR	0.68 (0.19–2.27)	0.520
Clinical PR	1.19 (0.24–6.16)	0.818
Virological CR	1.48 (0.28–9.26)	0.607
Virological PR	1.96 (0.57–6.28)	0.301
EBV-T cell	0.53 (0.10–1.68)	0.249
EBV-NK cell	0.56 (0.09–3.52)	0.543
EBV-T/NK cell	0.38 (0.05–2.95)	0.355

*CI* confidence interval of ratio, *HLH* hemophagocytic lymphohistiocytosis

## Discussion

CAEBV is an EBV-associated lymphoproliferative disorder that affects T and NK cells. It is related to some manifestations, including severe mosquito bite allergy, hydroa vacciniforme, or HLH [14]. CAEBV is a rare disorder and occurs frequently in East Asia for unknown reasons, easily progressing into extranodal NK/T lymphoma or aggressive NK cell leukaemia [15]. CAEBV is characterized by clonal proliferation of EBV-positive T and/or NK cells, followed by a dismal prognosis and resulting in prolonged or recurrent IM-like symptoms (e.g., fever, node swelling). Recently, the underlying mechanisms of CAEBV have gradually become clear [7]. EBV infection of T cells or NK cells can occur with a high EBV load in blood. In addition, cytotoxic T cells decrease in number or show dysfunction in CAEBV [7]. These findings suggest that undetermined immunosuppressive disorders might underlie persistent infection of T or NK cells, similar to the pathogenesis of HLH. In 2020, some new somatic mutations in host cells were confirmed to be related to the pathogenesis of CAEBV, such as DDX3X, KMT2D, BCOR, KDM6A and TP53 [16].

The CAEBV “3-step therapy” proposed by Japanese scholars has improved the overall survival rate. However, the remission rate of CAEBV disease activity before HSCT in "the 3‑step strategy” was only 20 to 30% [6]. In addition, there are other chemotherapies for bridging HSCT to treat CAEBV; however, the effectiveness of these options has been unsatisfactory, especially without virological CR, and have not agreed with each other [1]. The development of an effective treatment is urgently needed. Recently, Wang used the L-DEP regimen to treat refractory EBV-HLH, and 85.7% of the patients achieved an overall response [8]. PEG-asparaginase can attack EBV-infected T and NK cells, which might not be able to synthesize L-asparagine themselves [8, 17]. In EBV-infected cells, PEG-asparaginase induces hydrolysis of L-asparagine (essential amino acids for protein syntheses), thus preventing these cells from synthesizing the corresponding proteins, ultimately inhibiting cellular proliferation and resulting in a decline in EBV-DNA [17]. An in vitro study by Jinta et al. [18] demonstrated that L-asparaginase dose-dependently reduces the number of EBV-positive T and NK cells while not affecting the peripheral blood mononuclear cells of normal donors, suggesting the effect of the inhibition of L-asparaginase on the proliferation of EBV-positive T cells and NK cells [8, 18]. Therefore, the EBV DNA load in blood could be decreased because of blood cells being removed by treatment but not the clearance of EBV itself. Thus, it was of great interest to explore the role of the L-DEP regimen in treating CAEBV.

In this study, we found that the EBV-DNA loads in blood and plasma were significantly reduced by 8.1 times and 6.34 times compared with those at diagnosis, respectively, similar to the decrease by 10 times in serum 4 weeks after L-DEP therapy in EBV-HLH patients [9]. In addition, the clinical and virological response rates were up to 80% and 85.7%, respectively. Therefore, the L-DEP regimen was effective in reducing EBV-infected cells and EBV-DNA load in patients with CAEBV.

It has been reported that good clinical response is associated with good outcomes after allo-HSCT [1, 5]. In our study, we found that the clinical remission rate was 80.0% after L-DEP chemotherapy in CAEBV before bridging transplantation. This finding could have something to do with the introduction of PEG-asparaginase at the beginning of treatment. This result might be associated with good allo-HSCT outcomes [1, 5]. In addition, in this study, the 3-year OS after HSCT was 75.4%, lower than that with “the 3‑step strategy” [4]. This outcome might be attributed to the delay of HSCT due to the unavailability of suitable donors or economic reasons. Therefore, our results showed that L-DEP chemotherapy was sufficient for resolving disease activity before HSCT. However, to improve survival in patients with CAEBV, patients should undergo allo-HSCT as soon as possible after L-DEP chemotherapy to achieve better survival, and transplant-related mortality should be reduced.

Recently, acquired somatic mutations in DDX3X, KMT2D, BCOR, KDM6A and TP53 were confirmed to be associated with the pathogenesis of CAEBV [16, 19]. In this study, there were two patients with mutations in PIK3CD and IFNGR1. A heterozygous mutation was detected in PIK3CD in a patient, which was not detected in his parents. However, we only performed whole exome sequencing on mononucleated cells in peripheral blood, and we could not ascertain whether this mutation existed in other tissues. Thus, it was regarded as a spontaneous mutation. Whether these mutations were pathogenic factors of CAEBV is unknown. Although the PIK3CD mutation c.3061G > A (this mutation was not found in the normal population) detected in one of our patients has not been reported to be a pathogenic site of CAEBV, the gain-of-function mutation of PIK3CD was confirmed to be related to the pathogenesis of activated PI3 kinase delta syndrome (APDS), which is a primary immunodeficiency and can lead to EBV infection [20, 21]. Based on the patient’s history of infection, cellular immunodeficiency, and high pathogenicity predicted by SIFT and Polyphen2 software, we speculated that it could be a gain-of-function mutation. However, this speculation should be verified by in vitro and in vivo experiments, which will be undertaken in our future study. To date, the binding of IFN-g to INFGR1 has been confirmed to activate the JAK–STAT pathway, and IFNGR1 is an upregulated gene in patients with CAEBV. To date, more than 40 different mutations have been found in all 7 exons of the IFNgR1 gene, mainly leading to IFN-γ R1 deficiency [22]. Regarding the two IFNGR1 mutations detected in this study, the population mutation rates were 0.00020 and 0.00220, respectively. Since these two mutations were detected in the patient’s parents, they were germline mutations. Although IFNGR1 aberration has been reported to cause immunodeficiency 27A and tuberculosis infection, the function of these two mutations in CAEBV remains to be confirmed.

Until now, the factors related to poor prognosis in CAEBV were not clear. Kimura et al. reported that patients with T cell-type CAEBV had a poorer prognosis than those with NK cell-type CAEBV [23]. The prognosis of the childhood-onset group was better than that of the adolescent/adult and elderly onset groups [1]. However, Keisuke et al. showed that the type of infected cell or number of EBV-DNA copies in blood was not a prognostic factor [24]. In this study, we did not observe any factors associated with poor prognosis. This outcome could be due to fewer patients or insufficient follow-up time since slow progression is common in CAEBV. In future studies, more CAEBV cases and longer follow-up times are needed.

## Conclusion

In summary, our findings suggest that patients with CAEBV achieved a higher clinical remission rate and virological remission rate after L-DEP regimen treatment, that the EBV-DNA load in blood or plasma could be significantly reduced by sixfold, and that the enlarged liver and spleen also shrank with this regimen. Therefore, the L-DEP regimen is an effective therapy in CAEBV for bridging allo-HSCT. Although this study was small, we have provided some clinical evidence for the efficacy of the L-DEP regimen in CAEBV patients. Our current prospective and large-scale clinical trial will further assess the L-DEP regimen for patients with CAEBV.

## Data Availability

The data that support the findings of this study are available on request from the corresponding author.

## Abbreviations

EBV: Epstein–Barr virus
IM: Infectious mononucleosis
HLH: Hemophagocytic lymphohistiocytosis
CAEBV: Chronic active Epstein–Barr virus infection
EBER: Epstein–Barr virus-encoded small RNA
L-DEP: PEG-Aspegaspargase, doxorubicin, etoposide and methylprednisolone
CTL: Cytotoxic T cell
LMP: Latent membrane protein
VEGF: Vascular endothelial growth factor
JAK–STAT: Janus kinase–signal transducer and activator of transcriptions
IL: Interleukin
NF-κB: Nuclear factor κB
HSCT: Hematopoietic stem cell transplantation
